# Management of Ablation‐Refractory Atrial Fibrillation During Pregnancy

**DOI:** 10.1155/carm/1768682

**Published:** 2026-01-05

**Authors:** Faris Abu Za’nouneh, Kevin Benavente, Jadon Neuendorf, Blake Kadomoto, Christina Chong

**Affiliations:** ^1^ Department of Medicine, John A. Burns School of Medicine, University of Hawai’i, Honolulu, 96813, Hawaii, USA

**Keywords:** ablation, antiarrhythmics, atrial fibrillation, pregnancy, recurrence

## Abstract

Development of new atrial fibrillation (AF) is uncommon in pregnancy with an incidence of 0.03%. The occurrence of drug‐refractory AF during pregnancy in individuals with structurally normal hearts is even rarer, and there are no reports of ablation‐refractory AF during pregnancy in the literature. Early recognition and treatment of AF are crucial, as it causes hemodynamic changes that can adversely impact both the mother and fetus, potentially resulting in poor outcomes. We report a case of a 35‐year‐old woman at 19 weeks of gestation who presented with recurrent paroxysmal AF. Two years earlier, during her previous pregnancy, she was hospitalized for AF, where she experienced labetalol‐related hypotension and failed therapy with verapamil and digoxin. She was then managed with flecainide pill‐in‐pocket therapy until delivery. Eighteen months before this pregnancy, she underwent catheter ablation and remained stable until this current pregnancy, when she experienced 2 episodes of AF. These episodes were unresponsive to her scheduled regimen, prompting an increase in her beta‐blocker dose. However, the patient developed syncope and persistent palpitations prompting her presentation to the emergency department (ED).

## 1. Introduction

Pregnancy increases physiologic and metabolic demands, upregulates sympathetic tone and catecholamine release, significantly expands intravascular volume, and leads to a cascade of hormonal changes that induce cardiac stress. Arrhythmias are the most common cardiovascular complication in pregnant patients with or without pre‐existing heart disease [1]. While the overall incidence of developing AF during pregnancy is rare, its prevalence is rapidly growing, with an 111% increase in cases from 2000 to 2012 [2]. Historically, supraventricular tachycardias (SVTs) were the most common arrhythmias of pregnancy, but AF has surpassed SVT in prevalence, emerging as the leading arrhythmia identified. Contributors to the increased frequency of AF could be due to increases in maternal age and in risk factors such as hypertension, diabetes mellitus, and obesity in pregnancy [2, 3]. Additionally, women with a pre‐existing history of AF have a recurrence rate of up to 39.2% during pregnancy [4]. While 75% of AF cases during pregnancy will convert to normal sinus rhythm with medications alone, up to 12.3% will remain recurrent, permanent, or require direct current cardioversion [5]. Our case is of particular interest given that it highlights refractory, recurrent atrial fibrillation (AF) during pregnancy in a structurally normal heart. It underscores the challenges of pharmacologic management in pregnancy where titration is limited by maternal hemodynamic tolerance and fetal safety consideration, while highlighting the feasibility of invasive rhythm control strategies prior to delivery. We herein report a case of a 35‐year‐old pregnant woman presenting with recurrent symptomatic AF during the second trimester, refractory to multiple rate‐ and rhythm‐control therapies.

## 2. Case Report

**Figure 1:**
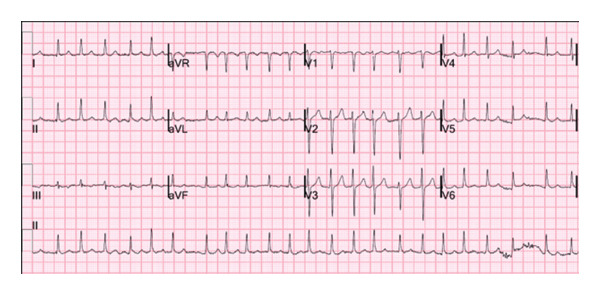
ECG during the patient’s 3rd pregnancy and hospitalization, demonstrating AF with a rapid ventricular rate.

**Figure 2:**
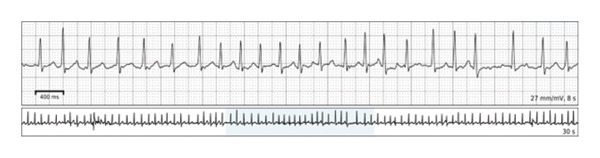
Ambulatory remote ECG monitoring after successful delivery of her 3rd pregnancy. The recording was obtained during an attempted repeat pregnancy that resulted in early fetal demise, 1 year prior to presentation. The rhythm strip demonstrates AF with a rapid ventricular rate.

A 35‐year‐old woman, Gravida 4 Para 1, at 19 weeks of gestation, presented to the ED with persistent palpitations, similar to her prior episodes of AF. The patient took her scheduled metoprolol succinate 50 mg without relief, prompting her to take an additional dose of metoprolol tartrate 25 mg. Shortly after, she experienced an episode of orthostatic syncope. She reported chest heaviness and mild shortness of breath during the episode of palpitations but denied ongoing chest pain on presentation. There were no recent changes in her oral intake, physical activity, or signs of illness. The patient had a history of recurrent symptomatic AF, first diagnosed during her third pregnancy 2 years prior to presentation, at 24 weeks of gestation. Despite being prescribed scheduled metoprolol and as‐needed flecainide, her AF was poorly controlled throughout her pregnancy, and she also failed trials of both digoxin and verapamil (Figure 1). She experienced recurrent symptoms throughout her pregnancy that continued after her successful delivery at 40 weeks of gestation. She later underwent a postpartum electrophysiology study, which was positive only for dual atrioventricular node pathway physiology, followed by catheter‐directed radiofrequency pulmonary vein isolation. She remained asymptomatic after the procedure until she became pregnant 1 year prior to this presentation. She again experienced an increased frequency of symptoms, correlated to AF, with rates up to 146 beats per minute as detected on her wearable electrocardiogram (ECG) monitoring device (Figure 2). She ultimately experienced an early pregnancy loss and elected to undergo misoprostol‐induced abortion.

Following the procedure, her palpitations and AF burden were noted to have significantly improved. However, her AF reemerged during the second trimester of her fourth pregnancy, experiencing two episodes of AF in the weeks leading up to her current admission. These episodes were resistant to her as‐needed flecainide, prompting her cardiologist to increase her daily metoprolol succinate dose from 25 to 50 mg, with the addition of metoprolol tartrate 25 mg as needed.

Her past medical history included two prior miscarriages. She denied any family history of cardiac issues, including AF, and has no history of smoking, drug use, or alcohol consumption during pregnancy.

**Figure 3:**
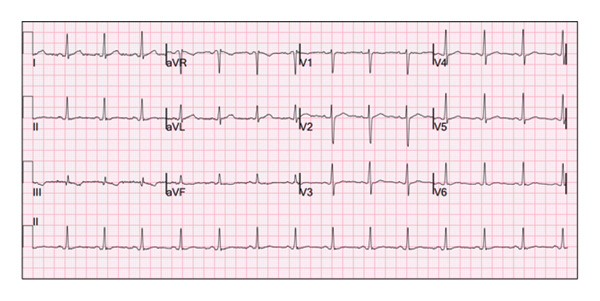
ECG from the patient’s current (4th) pregnancy showing a normal sinus rhythm with PVC and nonspecific T‐wave abnormalities.

**Figure 4:**
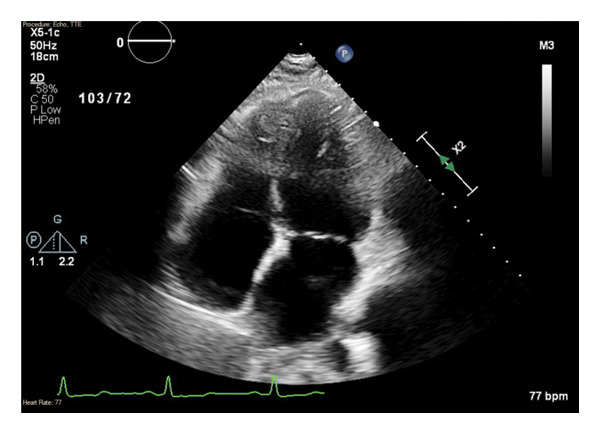
Echocardiogram on hospital presentation from the apical 4‐chamber view, demonstrating normal structure and function.

Laboratory workup was unremarkable including normal thyroid function and Troponin T. An ECG on admission showed sinus rhythm with premature atrial contractions (PACs) and nonspecific T‐wave abnormalities, as shown in Figure 3. Transthoracic echocardiogram (TTE) revealed normal left ventricular (LV) function (EF 55%–60%) without significant valvular abnormalities or chamber enlargement, consistent with normal prior imaging (Figure 4). An obstetric ultrasound confirmed a viable intrauterine pregnancy at 19 weeks and 3 days, with no placental or fetal abnormalities.

The patient was restarted on a lower dose of metoprolol succinate 25 mg daily, along with scheduled flecainide 75 mg twice daily. The patient was discharged, eventually undergoing outpatient loop recorder implantation (ILR), with tentative plans for repeating zero‐fluoroscopic catheter ablation prior to delivery. However, the patient was able to make it to term without invasive interventions, giving birth to a healthy infant. Her symptoms improved following delivery, remaining stable, and allowing her flecainide dose to be reduced to 50 mg twice daily, with no recurrent AF episodes noted on ILR. The patient had concerns about proceeding with ablation while breastfeeding and elected to defer the procedure to a later time.

## 3. Discussion

The rising prevalence of AF in pregnancy is noteworthy, as it increases the incidence of maternal mortality by over 10% (odds ratio: 13.1) and low birth weight by 21% [3, 6, 7]. Risk factors for the development of AF in pregnancy include rheumatic and congenital heart disease, advanced maternal age, and the typical cardiometabolic comorbidities in the general population. However, the strongest risk factor is a preexisting history of AF (odds ratio: 7.1) [6]. This was the key comorbidity of our patient, who developed recurrent episodes of AF, repeatedly exacerbated by her multiple pregnancies, despite having a structurally normal heart and no other pertinent medical history. Furthermore, the fact that her AF only flared during pregnancy, despite having undergone pulmonary vein isolation and having no structural heart disease, highlights the tremendous impact—pregnancy alone can exert on cardiac conduction. A complex combination of hormonal, autonomic, metabolic, and physiologic changes may synergistically contribute to the development of AF during pregnancy. Estrogen causes androgenic hypersensitivity increasing susceptibility to AF. Also, there is an increase in LV diameter and LV hypertrophy, leading to left atrial (LA) stretch, which increases the pressure and results in atrial electromechanical decoupling, predisposing to the development of arrhythmias during pregnancy [4].

The 2023 Heart Rhythm Society (HRS) expert consensus statement on the management of arrhythmias during pregnancy recommends direct current cardioversion for acute AF with hemodynamic compromise [8]. For stable patients with rapid ventricular rate (RVR), beta‐blockers are first‐line, with digoxin and calcium channel blockers serving as second‐line options safely in pregnancy. However, in our patient, the hemodynamic effects of the increased beta‐blocker dose resulted in an orthostatic syncopal event, necessitating the consideration of alternative agents. In addition, the lowest possible dose of beta‐blockers should be used, given the association with low birth weight, bradycardia, and neonatal hypoglycemia [8].

In cases of persistent or unstable AF, pregnant patients who are intolerant or unresponsive to rate‐control medications may safely undergo elective cardioversion without risking harm to the fetus. Additionally, if patients are having continued symptoms or RVR despite attempts at a rate control strategy, a pharmacologic rhythm‐control approach with the use of ibutilide, sotalol, or flecainide is a reasonable and pregnancy‐safe option in the absence of structural heart disease. Amiodarone is a last resort and is often avoided due to multiorgan fetal toxicities [8]. In our patient, her paroxysmal AF was not effectively controlled with an as‐needed flecainide rhythm‐control strategy, and she was switched to scheduled flecainide while invasive procedural options were considered.

Although the data on flecainide use during pregnancy are somewhat limited, growing clinical experience, including findings from multiple retrospective reviews, as well as from our study, suggests that it can be a safe and effective option when used appropriately. It is generally reported as nonteratogenic, though fetal or neonatal QRS widening has been described with prolonged exposure [8]. In our case, flecainide was part of a rhythm‐control approach in a pregnant patient with paroxysmal AF who continued to experience symptoms despite attempts at rate control. Initially managed with an as‐needed regimen, her symptoms improved after switching to scheduled flecainide dosing, while procedural options were being considered. This supports the idea that in cases where AF is persistent or poorly tolerated, especially when rate‐control medications are ineffective or not well‐tolerated, flecainide offers a reasonable and pregnancy‐safe alternative, provided there is no underlying structural heart disease. Other studies echo these findings, particularly in the setting of Wolff–Parkinson–White (WPW) syndrome, where flecainide has been shown to prevent dangerous arrhythmias by blocking accessory pathways, without causing harm to the fetus [9]. Compared to catheter ablation, which carries procedural risks during pregnancy, flecainide stands out as a noninvasive, well‐tolerated option especially in centers without high procedural volumes. Overall, our experience adds to the growing reassurance that flecainide, when used thoughtfully, is a safe and effective tool in managing challenging arrhythmias during pregnancy [9].

Regarding ablation, multiple studies have demonstrated that fetal exposure to intraprocedural radiation is minimal and can be further reduced or eliminated with the use of advanced 3D mapping technology. Additionally, maternal rate and rhythm control should be prioritized over the potential risk of fetal radiation exposure [8]. Both fetal and maternal outcomes following AF ablation during pregnancy have demonstrated both safe and efficacious results [10], thus reinforcing the notion that, while a careful multidisciplinary risk‐benefit discussion between cardiology, maternal–fetal medicine, obstetrics, and the patient is essential, pregnant patients have a wide range of treatment options that can be safely utilized, even in cases of resistant arrhythmias. In the setting of a planned redo ablation, reassessing pulmonary vein isolation durability, coordinating anticoagulation, and recognizing the heightened impact of complications such as pericardial effusion, stroke, and bleeding are key considerations. Although the patient deferred the procedure due to breastfeeding, these factors illustrate the complexity of planning repeat ablation during pregnancy.

Our case highlights the complex management of treatment‐resistant AF during pregnancy, particularly when initial ablation attempts fail, and how a repeat ablation can be a reasonable and relatively safe option. Furthermore, pharmacologic management of AF during pregnancy must balance arrhythmic efficacy with safety to both the fetus and the mother. This case underscores the importance of further research to guide and identify optimal treatment approaches for arrhythmia during pregnancy.

## Ethics Statement

The authors have nothing to report.

## Consent

A written informed consent was obtained from the patient prior to reporting this case.

## Disclosure

All authors read and approved the final version of the manuscript.

## Conflicts of Interest

The authors declare no conflicts of interest.

## Author Contributions

Faris Abu Za’nouneh contributed to the conception, drafting, and writing of the manuscript. Kevin Benavente, Jadon Neuendorf, Blake Kadomoto, and Christina Chong assisted with data collection, literature review, writing, and editing of the manuscript.

## Funding

No funds were received.

## Data Availability

The experimental data used to support the findings of this study are available from the corresponding author upon reasonable request.
